# Pan-cancer analysis of TRPV1: a novel immune infiltration-related biomarker for tumor prognosis and immunotherapy response prediction

**DOI:** 10.1186/s12885-026-15576-4

**Published:** 2026-01-14

**Authors:** Feixiang Wang, Boyun Deng, Maohua Qin, Zijin Cheng, Jian Zhang, Guofeng Xie

**Affiliations:** 1 Department of Thoracic Surgery, Guangzhou Institute of Cancer Research, The Affiliated Cancer Hospital, Guangzhou Medical University, Guangzhou, China; 2 Department of Thoracic Surgery, Affiliated Hospital of Guangdong Medical University, Zhanjiang, China; 3 Department of Radiation Oncology, Guangzhou Institute of Cancer Research, the Affiliated Cancer Hospital, Guangzhou Medical University, Guangzhou, China; 4 School of Life Science Fudan University, Shanghai, China

**Keywords:** TRPV1, Prognosis, Single-cell, Tumor microenvironment, Pan-cancer

## Abstract

There were several studies on Transient receptor potential vanilloid 1 (TRPV1), the research on TRPV1 in cancer remains unclear. The expression, prognostic, single-cell value, immune cell infiltration, and immune check-point (ICI) of TRPV1 in pan-cancer were assessed. Functional assays were performed to evaluate the effect of TRPV1 in vitro. TRPV1 was significantly upregulated in 13 cancers, and was closely correlated with poor prognosis. Moreover, the expression of TRPV1 was significantly correlated with the infiltration of immune cells and ICI. Cellular function and western blot experiments revealed that TRPV1 may be required for cancer cell migration in head and neck cancer and lung cancer by inhibition of PI3K/AKT and promotion of immune cell infiltration and autophage. Our study suggests that TRPV1 may be able to regulate the tumor microenvironment by regulating immune-related physiological activities and thereby regulating the occurrence and progression of cancers. Thus, TRPV1 could be used to develop new-targeted drugs for cancers.

**Supplementary Information:**

The online version contains supplementary material available at 10.1186/s12885-026-15576-4.

## Introduction

Cancer is the leading cause of death worldwide and a major impediment to extending life expectancy [1]. An estimated 19.3 million new cancer diagnoses and about 10.0 million cancer deaths worldwide occurred in 2020 [2], with few effective cancer therapies available thus far. Cancer immunotherapy, particularly immune check-point inhibitors, has emerged as one of the most promising therapeutic methods in recent years [3–5]. The tumor microenvironment (TME), on the other hand, has been widely investigated in the cancer literature, with a particular emphasis on its involvement in cancer occurrence and progression [6–8], as well as its potential as a cancer therapeutic target [9, 10].

Tumor cells and their surrounding immune cells, tumor-related fibroblasts, vascular endothelial cells, and other cells comprise TME [11–13]. These cellular connections allow tumor cells to avoid immune detection and constitute the cytological basis of tumor development and metastasis [14, 15]. Innate immune cells, including, macrophages, neutrophils, natural killer cells, dendritic cells, and bone marrow-derived suppressor cells and adaptive immune cells, such as, T cells and B cells are important components of the TME and are involved in oncogenesis and tumor progression. Thus, it is critical to understand the tumor microenvironment in order to identify important biomarkers associated with predicting response to immunotherapeutic therapies.

Transient receptor potential vanilloid 1 (TRPV1), a nonselective cationic channel, is involved in the Ca^2+^ influx. TRPV1 is found in a variety of organs, particularly the brain and kidney and it is involved in not just pain perceptions but also other biological processes such as autophagy, inflammation and apoptosis [16, 17]. Furthermore, TRPV1 has been proposed as a possible target for cancer therapy since it was shown to be aberrantly expressed and foster resistant features in a variety of different types of tumors [18, 19]. However, the role of TRPV1 in regulating TME remains unclear.

In this study, we comprehensively explored the genetic and genomic aspects of TRPV1 and its relationship to the prognosis in pan-cancer. We further evaluated the relationship between the expression of TRPV1 and immune check-point regulators, TMB, MSI, and immune response. In addition, we also evaluated the pathways enriched by TRPV1 in cancers. Our findings shed novel light on TRPV1’s functional involvement in pan-cancer, suggesting potential strategies by which TRPV1 influences tumor development via modulating the TME.

## Methods and materials

### Data sources and processing

The mRNA expression data and the clinical data for The Cancer Genome Atlas (TCGA) and Genotype-Tissue Expression (GTEx) were downloaded from the UCSC XENA database (https://xenabrowser.net/datapages/). The genomic and epigenetic alteration of TRPV1 was obtained from the cBioPortal database (https://www.cbioportal.org/). Data on TRPV1 modifications (amplifcation, mutation, structural variation, deep deletion, and multiple alterations) matched with 30 cancer types were acquired from “Cancer Type Summary” items.

### Differential expression analysis of mRNA

Gene expression data of pan-cancer containing paired normal samples was downloaded from TCGA to analyze the differentially expressed genes between tumors and normal tissues. Then, the fold change and adjusted p-value were calculated by the “limma” package. Finally, we defined genes with an adjusted P-value less than 0.05 as differential expression genes (DEGs).

### Prognostic analysis

Kaplan–Meier and Univariate Cox regression analysis were performed to assess the overall survival (OS), disease-free survival (DFS) of patients with pan-cancer. The GEPIA2 database (http://gepia2.cancer-pku.cn/#index) was used to explore the correlation analysis between copy number variation and clinical survival and validate the prognostic value of the OS of TRPV1in cancer [20].

### Single-cell analysis of TRPV1

Tumor Immune Single-cell Hub (TISCH, http://tisch.comp-genom ics.org/) was used to further estimate the correlation of “TRPV1” expression in the TME at a single-cell resolution [21]. TISCH is a scRNA-seq database that focuses on the TME. TISCH offers comprehensive cell type annotations, including immune cells, stromal cells, and malignant cells at the single-cell level, allowing for the analysis of TME across cancer types.

### Gene Set Enrichment Analysis (GSEA)

GSEA analysis with “TRPV1” was conducted. The Hallmark gene set file was downloaded in “gmt” format from the Molecular Signatures Database (MSigDB, https://www.gesa-msigdb.org/gsea/index.jsp). GSEA analysis was performed with the R packages “clusterProfiler” and “GSVA” to calculate the standardized enrichment score (NES) and error rate (FDR) for each cancer type. The results were summarized in the R package “ggplot2” visualization and the bubble diagram was depicted.

### TME analysis of TRPV1

To explore the roles of TRPV1 in infltrating cells and the TME, the identified immune cells were then verified in the Tumor Immune Estimation Resource 2.0 database (TIMER 2.0) with the ‘Immune_Gene module‘ [22]. We used several methods, such as TIMER, EPIC, QUANTISEQ, XCELL, MCP-COUNTER, CIBERSORT, and CIBERSORT-ABS, to identify immune cells that correlated strongly in the same directions. The TIMER2.0 correlation module was used to investigate the relationships between the indicators of substantial tumor-infiltrating immune cells and TRPV1 expression.

### Immune Check-point Inhibitor (ICI) cohort validation

The correlation of TRPV1 and 47 immune check-point regulators was explored based on the Sangerbox website (http://sangerbox.com). Spearman’s correlation test was used to investigate correlations between TRPV1 mRNA expression and microsatellite instability (MSI) or tumor mutation burden (TMB). The immunotherapy value of TRPV1 was analyzed in the IMvigor210 immunotherapy cohort.

### Cell culture and ShRNA transfection

Two head and neck squamous cell carcinoma cell lines (HSC-3 and CAL-27) were obtained from ATCC (American Type Culture Collection) and cultured in Dulbecco’s Modified Eagle Medium (DMEM; Gibco, Beijing, China) supplemented with 10% fetal bovine serum (FBS; Gibco, Australia) at 37 °C in a 5% CO2 humidified incubator. Cells were plated into a 6-well plate at a density of 70–90%. The specific shRNA sequence used to target TRPV1 is GCAGACACTGGAACTTTGTTCAA, and its corresponding scrambled sh-RNA control (sh-NC) were obtained from Vigene Biosciences (Rockville, USA).

### Colony formation and wound − healing assay

In 6-well plates, 1000 cells were seeded per well for 14 days. Colonies were counted after staining with 0.2% crystal violet for 10 min. In 6-well plates, at a density of 1*10^5^ cells transfected with sh-TRPV1 and sh-NC per well were seeded. A straight linear wound was made in each well by using a 1 mL pipette tip. Then, the cells were carefully washed with PBS and cultured in DMEM supplemented with 5% FBS. Finally, wound healing images were taken at 0 and 36 h by using an inverted microscope with a 200x objective.

### Transwell assay

Cells transfected with sh-TRPV1 and sh-NC at a density of 1*10^5^ cells/well were inoculated above the 8 μm chamber (Corning Inc., NY-Corning, USA) with a medium containing serum-free medium and below the chamber with a medium containing 10% FBS. Cells that invaded or migrated into the lower compartment were counted after 24 h with 0.2% crystal violet.

### Western blot analysis

Cells were washed twice with phosphate-buffered saline (PBS) and lysed in RIPA buffer supplemented with a cocktail of protease and phosphatase inhibitors (Sigma-Aldrich). Lysates were clarified by centrifugation at 12,000 × g for 15 min at 4 °C to remove cellular debris. Equal amounts of protein were resolved by SDS-polyacrylamide gel electrophoresis (SDS-PAGE; Bio-Rad, Hercules, CA) and subsequently transferred onto polyvinylidene fluoride (PVDF) membranes (Bio-Rad). Membranes were blocked with 5% (w/v) nonfat dry milk in Tris-buffered saline containing 0.1% Tween-20 (TBST) for 2 h at room temperature, followed by overnight incubation at 4 °C with the appropriate primary antibody. After washing, membranes were incubated with a horseradish peroxidase (HRP)-conjugated secondary antibody, and immunoreactive bands were detected and quantified using a chemiluminescent western blot imaging system (Bio-Rad).

### Immunohistochemistry (IHC)

Paraffin-embedded primary tumor sections were deparaffinized and rehydrated through a xylene-based clearing step followed by a graded ethanol series. Antigen retrieval was carried out using a modified citrate buffer (pH 6.0). Endogenous peroxidase activity was blocked by incubating the sections with 3% hydrogen peroxide (H₂O₂) for 15 min at room temperature. The sections were then incubated overnight at 4 °C with the appropriate primary antibody (TRPV1 (abcam ab305299, 1:100), Arg1 (PTG 16001-1-AP, 1:200), CD8 (Zhongshan Jinqiao za-0508, prediluted antibody), LC3 (PTG 14600-1-AP, 1:300), PD-L1 (abcam ab213524, 1:100)). This was followed by a 1-hour incubation at room temperature with a biotin-conjugated goat anti-rabbit IgG secondary antibody. Finally, immunoreactivity was visualized using an avidin–biotin–peroxidase complex (ABC) detection system and a chromogenic substrate.

### Statistical analysis

The data were presented as means ± standard error (SD). Differences between groups analysis was performed by using the Wilcoxon rank sum test and the one-way ANOVA. Univariate Cox regression analysis and the Kaplan-Meier method were applied to calculate the prognosis role of TRPV1 in cancers. Statistical analysis was performed using R 3.6.3 and the spearman analysis was used as the correlation coefficient. *P* < 0.05 was considered statistically significant.

## Results

### The genomic and genetic alteration of TRPV1 in cancers

**Fig. 1:**
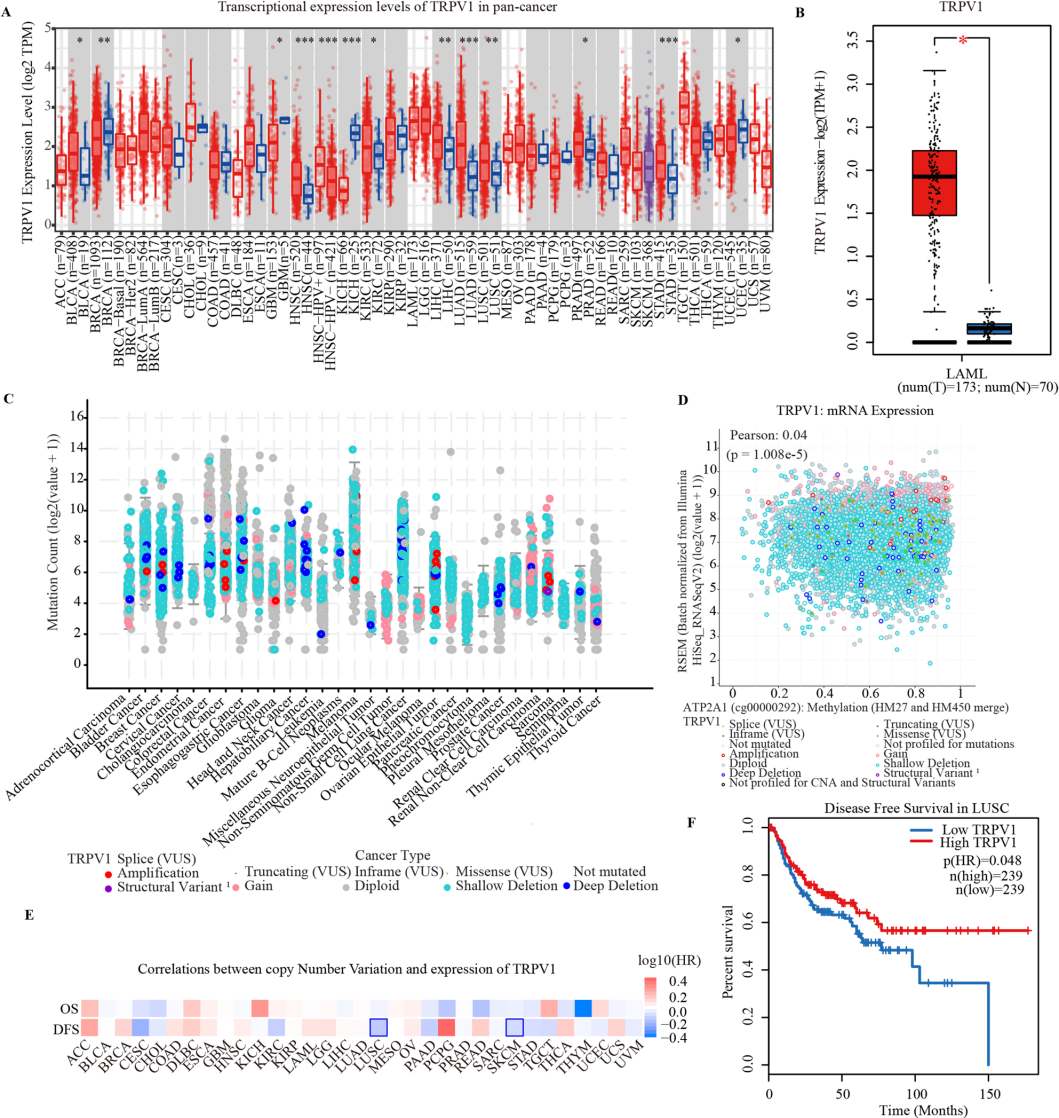
The genomic and genetic alteration of TRPV1 in cancers. **A** Transcriptional expression level of TRPV1 in pan-cancer. **B** The mRNA expression level of TRPV1 in LAML. **C** The genomic alteration of TRPV1 in pan-cancer. **D** Correlation analysis between genomic alteration and mRNA expression. **E** Correlation analysis between clinical survival and mRNA expression. **F** The disease-free survival of TRPV1 in LUSC. LAML, acute myeloid leukemia; LUSC, lung squamous cell carcinoma. The significance threshold was set as *P* < 0.05. *P < 0.05, **P < 0.01, ***P < 0.001

The expression of TRPV1 in TCGA pan-cancer was firstly analyzed. As shown in Fig. 1A, In the unpaired samples, TRPV1 was overexpressed in 13 tumors: bladder urothelial carcinoma (BLCA), breast invasive carcinoma (BRCA), glioblastoma multiforme (GBM), head and neck squamous cell carcinoma (HNSC), kidney chromophobe (KICH), kidney renal clear cell carcinoma (KIRC), liver hepatocellular carcinoma (LIHC), lung adenocarcinoma (LUAD), lung squamous cell carcinoma (LUSC), prostate adenocarcinoma (PRAD), stomach adenocarcinoma (STAD), and uterine corpus endometrial carcinoma (UCEC) (*P* < 0.05). And in the paired samples, the expression of TRPV1 was significantly upregulated in acute myeloid leukemia (LAML) (Fig. 1B). We further analyzed the genomic alteration of 30 cancers in which TRPV1 showed different mutation types in cancers, such as amplification, structural variant, gain, truncating, inframe, diploid, shallow deletion, deep deletion and missense. Shallow deletion was the main mutation type in most cancers (Fig. 1C). Correlation analysis indicated that there was a weak correlation between genomic alteration and mRNA expression (*R* = 0.04, *P* = 1.008e-5; Fig. 1D), while correlation analysis the mutation alteration, such as splice, truncating, inframe, missense, were also involved in mRNA expression of TRPV1 (*R* = 0.21, *P* = 9.29e-96 ; Figure S1). Survival analysis indicated that the dysregulation of TRPV1 was associated with clinical OS and DFS in cancers (Fig. 1E). The prognosis indicated that the low expression of TRPV1 was associated with poor DFS in LUSC (*P* = 0.048; Fig. 1F). Subgroup analysis indicated TRPV1 was associated T stage in ESCA, KIRP, KIPAN and MESO, N stage in PRAD, LIHC and ACC, M stage in CESC, LUAD, COADREAD and ACC, clinical stage in OV (Figure S2).

### Single-cell analysis of TRPV1 expression across various cell types

**Fig. 2:**
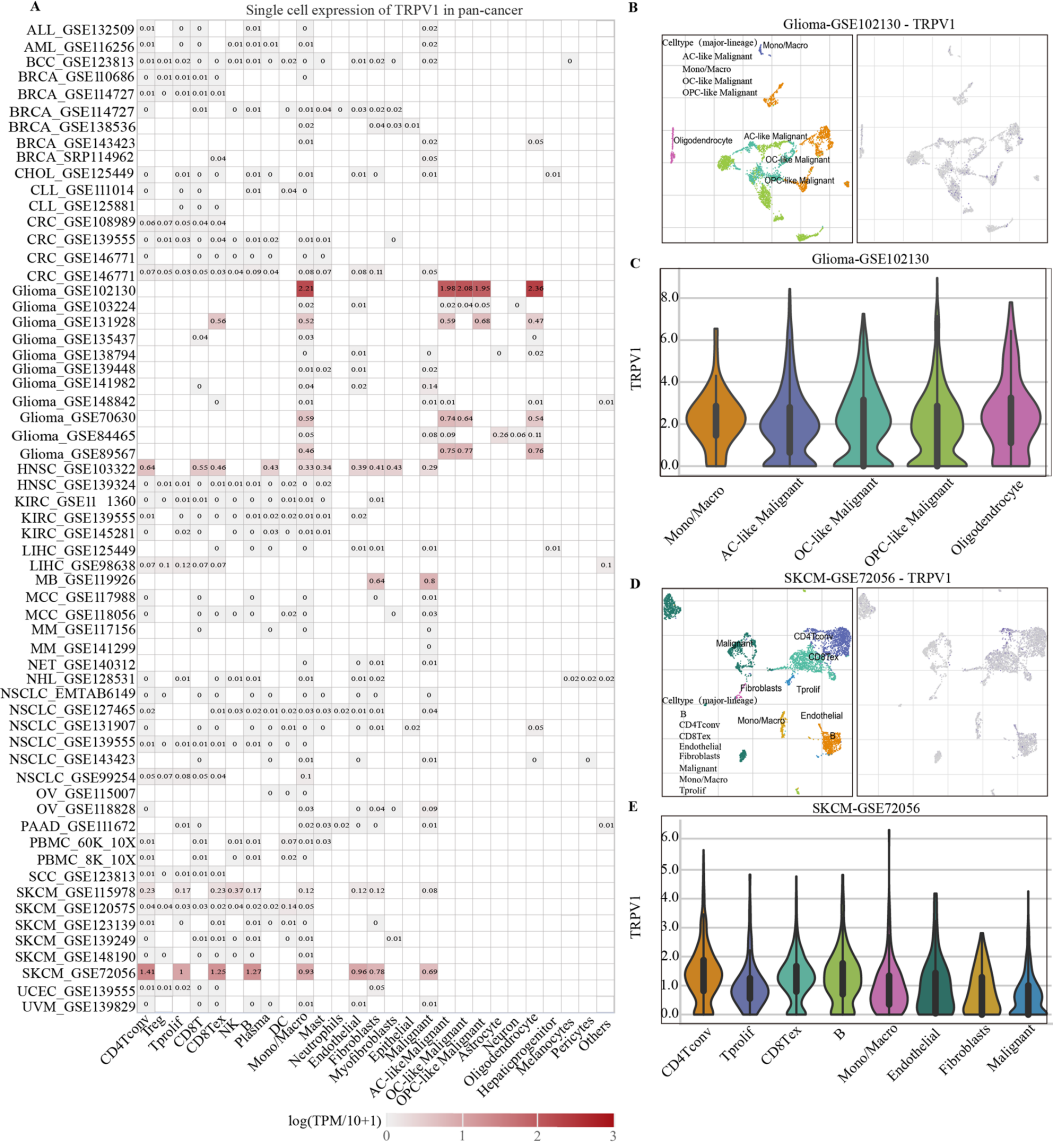
Single-cell scale TRPV1 expression atlas in pan-cancer cohorts. **A** TRPV1 expression values in cancer cohorts. **B**-**C** Main cell types expressing TRPV1 in the Glioma-GSE102130 cohort. **D**-**E** Main cell types expressing TRPV1 in the SKCM-GSE72056 cohort

To assess TRPV1 expression patterns in tumor heterogeneity, we used the TISCH tool to investigate individual TRPV1 expression in immune and malignant cell populations. TRPV1 expression was measured in each individual cell and then averaged (Fig. 2A). TRPV1 was shown to be most abundant in immune cells, notably T lymphocytes (CD4 Tconv, T reg, T prolif, CD8 T, CD8 Tex cells), followed by non-specific immune cells such as NK cells, DCs, monocytes, and macrophages. Notably, TRPV1 was mainly upregulated in malignant cells. TRPV1 expression was stronger in glioma (GSE102130, GSE131928, GSE70630, and GSE89567) and SKCM (GSE115978 and GSE72056). Specifically, we visualized TRPV1 expression in glioma (GSE102130) and SKCM (GSE72056) datasets, and the cell types with the highest TRPV1 expression were highlighted in Fig. 2B-E. The possible importance of TRPV1 in the TME of glioma and melanoma expands to its influence on immune cells.

### Correlation between immune cell infiltration and TRPV1 expression in cancers

**Fig. 3:**
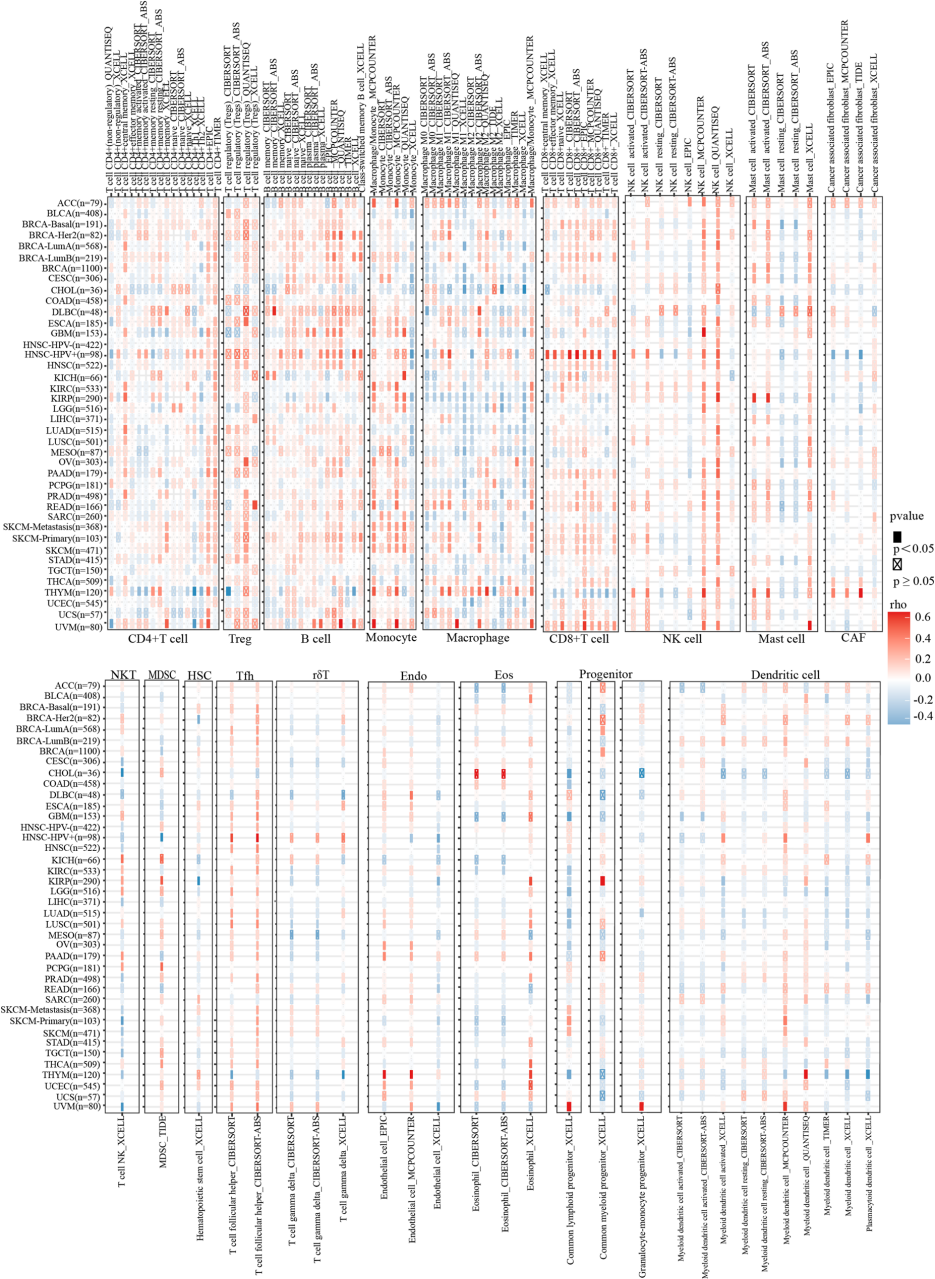
Correlation between TRPV1 expression and immune cell infiltration in pan-cancers. Correlations between TRPV1 expression and immune cell infiltration were analyzed using TIMER 2.0. Red and blue blocks indicate positive and negative correlations, respectively. *P* < 0.05 was considered significant

To know the role of TRPV1 in regulation infiltration of immune cells, we then assessed the possibility of a link between it and immune cell infiltration. In the pan-cancer cohort, spearman correlation analysis of the connection between TRPV1 expression and infiltration levels of several immune cell lineages was performed. As presented in Fig. 3 and Table S1, the results illustrated that the positive relationship between TRPV1 expression and several cell types, particularly Treg, B cells, monocyte and Tfh. Furthermore, positive relationships were confined mostly to the same cell lineages; that is, particular infiltrating cell types were positively connected with TRPV1 expression in a variety of cancer types. TRPV1 expression was shown to be positively connected with the infiltration of most immune cells studied, with the exception of several particular subtypes such as mast cells, dendritic cell and macrophages. These results revealed that correlations between TRPV1 expression and four specific immune cell sub-populations (Treg, B cell, monocyte and Tfh cells) infiltration levels were evident in pan-cancer cohorts.

### The pathways enriched by TRPV1 in cancers

Given TRPV1’s prognostic and immune infiltration in many malignancies, we studied the underlying biological processes or pathways connected with TRPV1 to better understand the probable mechanisms involved. Thus, we evaluated a hallmark gene collection, which is made up of marker genes that define tumor biological state and progression. The hallmarks gene sets between the high TRPV1 and low TRPV1 groups were enriched. As plotted in Figure S3 and Table S2, our findings demonstrated a highly concentrated distribution of enrichment across 33 pan-cancer types, with immune-related pathways being considerably enriched in tumors with high TRPV1 expression. TNF-a signaling via the NF-kapaB route, interferon-gamma response, interferon-alpha response, inflammatory response, PI3K/AKT/MTOR, and allograft rejection were among the enrichment pathways. TRPV1 was also shown to be abundant in the metastasis pathways, including epithelial-mesenchymal transition, apical junction, and angiogenesis. When it came to cancer types, BLCA, BRCA, CESC, cholangiocarcinoma (CHOL), colon adenocarcinoma (COAD), HNSC, LIHC, LUAD, LUSC, PAAD, PRAD, READ, STAD, THCA and UCEC had significant enrichment of the aforementioned pathways.

### Correlation between TRPV1 expression and the TME

**Fig. 4:**
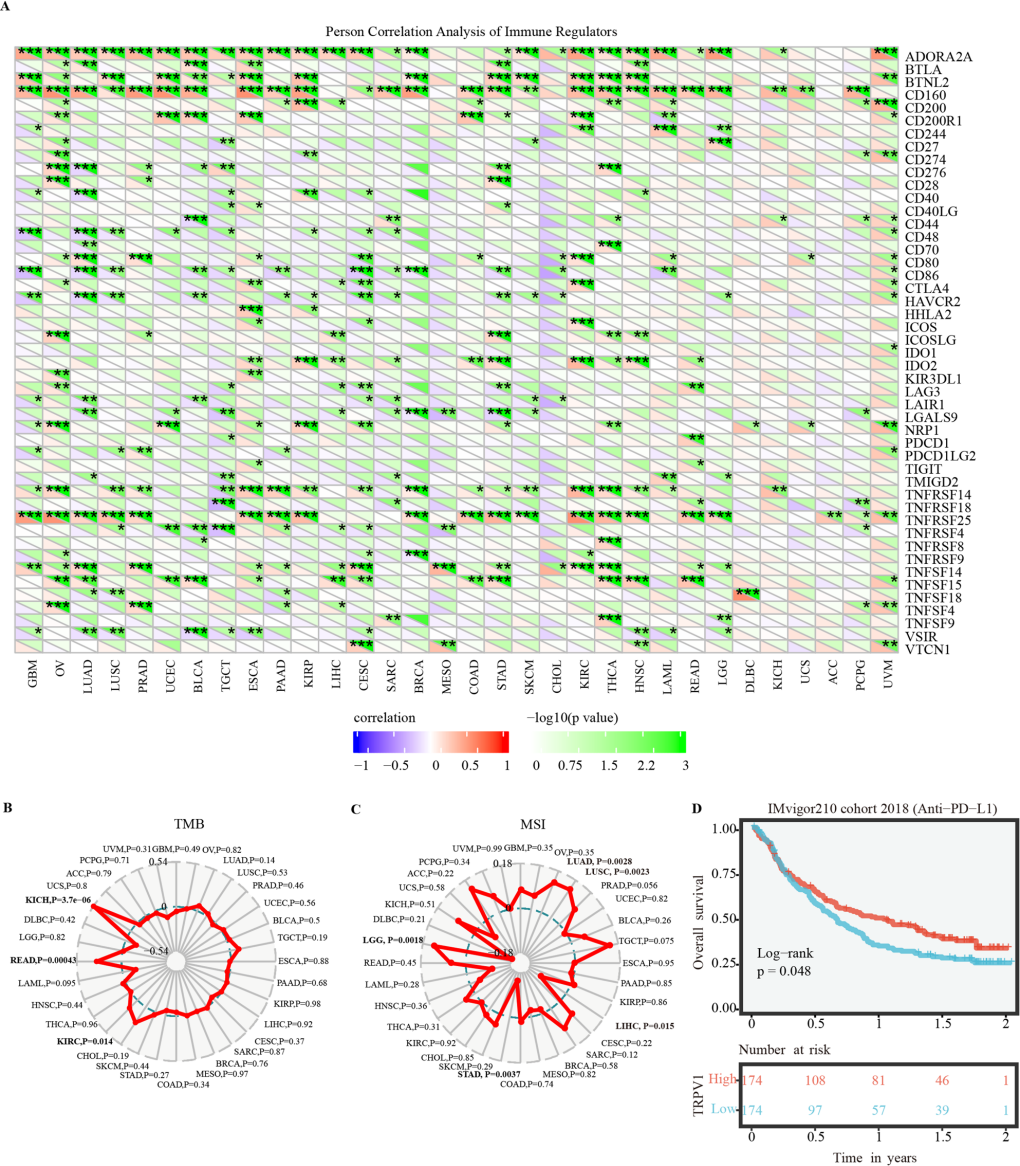
Correlations of TRPV1 expression with TME biomarkers and clinical responses to immunotherapy. **A** Heatmap showing correlations between TRPV1 expression and immune checkpoint regulators. **B**-**C** Correlations of TRPV1 expression with TMB (**B**) and MSI (**C)**. **D** Overall survival analysis of patients with high and low TRPV1 expression from patients with urothelial cancer receiving anti-PDL1 immunotherapy (IMvigor210 cohort)

Tumor cells typically avoid immunological assault by suppressing immune responses. As the methods previously reported [23], 47 immune check-point regulators were chosen and evaluated in the context of TRPV1 expression. TRPV1 expression and levels of individual immune check-point regulators were correlated using Spearman’s correlation analysis. Overall, our findings indicated a highly substantial positive connection. Pan-cancer analysis indicated that TRPV1 expression was positively correlated with immune regulators in various cancers, particularly OV, LUAD, LUSC, Testicular Germ Cell Tumors (TGCT), Esophageal carcinoma (ESCA), PAAD, Kidney renal papillary cell carcinoma (KIRP), STAD, KIRC, THCA, and HNSC. Regarding individual immune check-point regulators, correlations with TRPV1 in each cancer were highly significantly positive or negative; specifically, ADORA2A, BTLA, BTNL2, and TNFRSF25 exhibited markedly stronger correlations than other immune check-point regulators (Fig. 4A and Table S3).

To understand the role of TRPV1 in immunotherapy, the association between TRPV1 and immunotherapy-related biomarkers (TMB and MSI) was further assessed. As shown in Fig. 4A-B, TRPV1 expression was positively associated with the TMB in KICH (*P* = 3.7e-06), READ (*P* = 0.00043) and KIRC (*P* = 0.014); MSI in LUAD (*P* = 0.0028), LUSC (*P* = 0.023), LIHC (*P* = 0.015), STAD (*P* = 0.0037), and LGG (*P* = 0.018). The survival analysis IMvigor210 cohort indicated that groups with high TRPV1 expression had a higher OS probability and a longer OS time than those with low TRPV1 expression. These results indicated that the dysregulated expression of TRPV1 was associated with prognosis and immune cell infiltration, which may act as a molecular biomarker for predicting the immune response of cancer patients.

### TRPV1 promotes cancer cell growth and migration

**Fig. 5:**
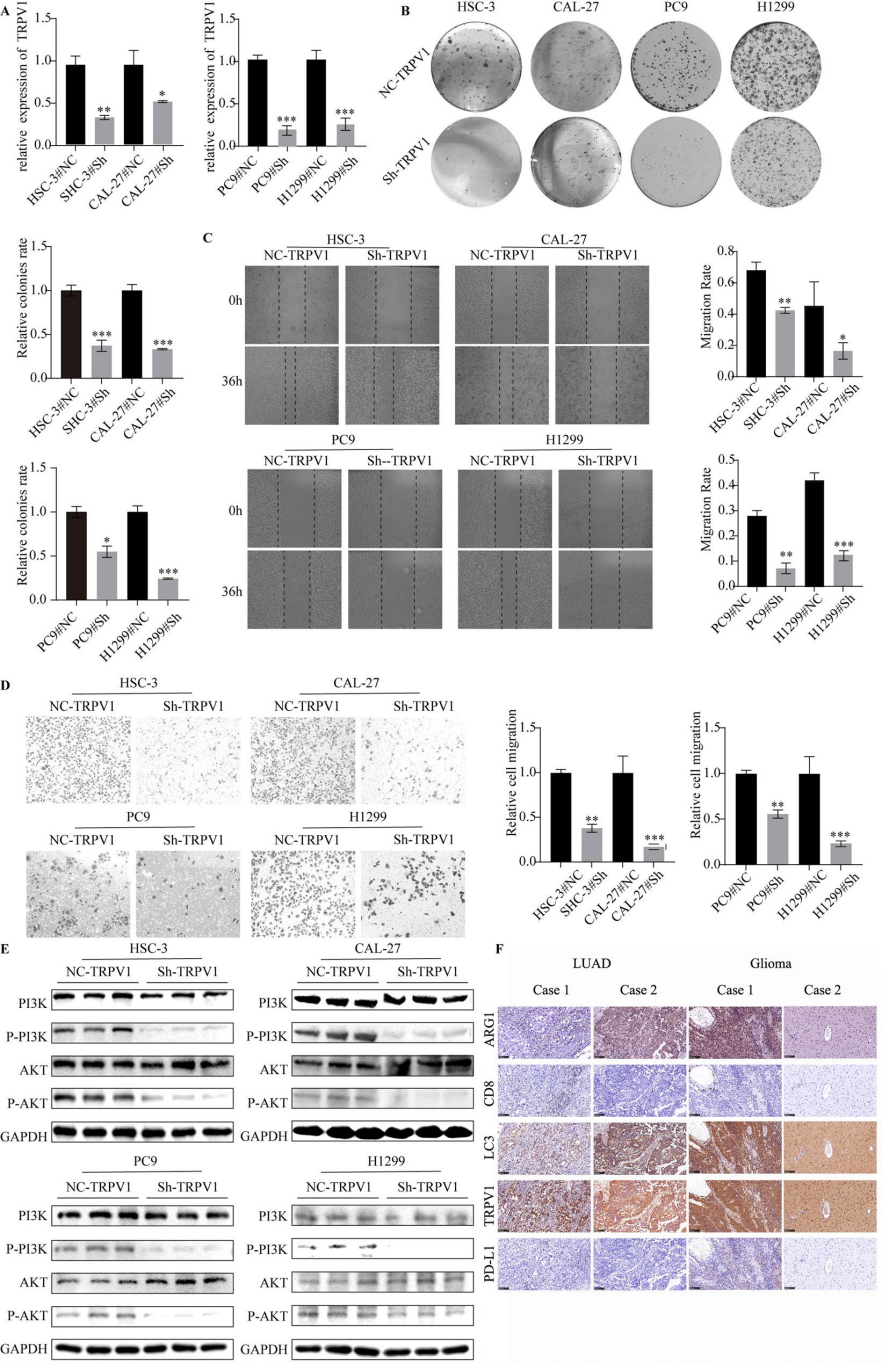
Knockdown of TRPV1 inhibited head and neck cancer cell proliferation and migration. **A** Analysis of TRPV1 expression in different head and neck cell lines by qRT-PCR. **B** The colony formation assays of different head and neck cell and lung cancer lines were analyzed. Scale bar: 100 μm. **C**-**D** A comparison of migration assays between TRPV1-NC and TRPV1-knockdown head and neck cell lines and lung cancer were performed. **E** The PI3K/AKT pathway detected by western blot. **E** The expression of Arg1, CD8, TRPV1, LC3 and PD-L1 detected by immunohistochemistry. *P < 0.05, **P < 0.01, ***P < 0.001

To identify the roles of TRPV1 in the development and progression of head and neck cancer, the downregulation head and cancer cell and lung cancer model were verified by qPCR (Fig. 5A). The colony formation assay results indicated that the anchorage-dependent growth of HSC-3, CAL-27 and PC9, H1299 cells were significantly inhibited when TRPV1 was knocked down (Fig. 5B). In the wound healing assays, siRNA-induced downregulation of TRPV1 led to a decrease in the wound healing rate (Fig. 5C). Consistent with these findings, transwell assays showed that the ability of cell migration and invasion in HSC-3, CAL-27 and PC9, H1299 cells were also attenuated when TRPV1 was knocked down (Fig. 5D). To identified the potental menchanism of TRPV1, the western blot showed that knockdown TRPV1 can significantly inhibit the activation of PI3K/AKT pathway (Fig. 5E). Immunohistochemical analysis of serial sections indicated that TRPV1 was positively associated with expression of CD8 and LC3, negatively associated with Agr1 (Fig. 5F).Taken together, knockdown of TRPV1 inhibited various malignant behaviors involved in head and neck and lung cancer progression.

## Discussion

ICI seem to be an effective and prospective treatment for cancer patients [24]. However, only a limited portion of patients show responses to currently available immunotherapy and the quest for new immune-associated targets and biomarkers is critical [25–28]. The results clearly demonstrated that TRPV1 RNA level was dysregulated among various tumor tissue types, except BRCA, CESC, CHOL, COAD, Lymphoid Neoplasm Diffuse Large B-cell Lymphoma (DLBC), KIRP, MESO, PAAD, PCPG READ, SKCM, THCA, and Uveal Melanoma (UVM) by plotting TRPV1 transcriptome data derived from the TCGA and GTEx database; however, previous studies have reported down-regulation of both TRPV1 mRNA and protein levels [7, 29, 30], which contradicts our findings. As a result, it is feasible to infer that there may be genetic and genomic alterations that mitigate the higher levels of TRPV1. TRPV1 gene changes were found at a frequency of 1.6% in the examined pan-cancer population, and mutation types were shallow deletion, amplification, and gain, indicating that they were likely to contribute significantly to tumorigenesis.

To explore the heterogeneity and complexity of tumors, the expression levels of TRPV1 in the cellular component were further evaluated. Single cell analysis indicated that TRPV1 was found in high concentrations in a variety of immune cells, most notably adaptive immune cells such as CD4 + and CD8 + T lymphocytes, followed by innate immunity cells such as monocytes, macrophages and DCs. The western blot found that knockdown TRPV1 was significantly with inhibition of PI3K/AKT pathway in head and neck cancer and lung cancer. Immunohistochemistry found that TRPV1 was positively associated with CD8, negatively associated with Agr1 by immunohistochemical analysis of serial sections.TRPV1 expression was also shown to be dispersed throughout all possible tumor cell lineages [31–33], which may clarify the contradictory context between transcriptional results and previous experimental studies to some extent.

Tumor development and progression rely on the adjacent TME, which also determines the effectiveness of immunotherapy [34]. In this study, we found TRPV1 not only mediated the infiltration of Treg, B cell, monocyte, and Tfh in TME, but also regulated the expression of immune check-point regulators, immune- and metastasis- related pathway. Consistent with the above results, TMB and MSI are reported biomarkers that can predict TME status and the anti-tumor efficacy of ICI therapy [35–37], and correlation analysis also identified the correlations of TMB and MSI with TRPV1 expression. TRPV1 was associated with good immune therapy responses in the IMvigor210 cohort. These results indicated that TRPV1 is a promising biomarker for predicting cancer prognosis and a molecular target for improving clinical immune therapy.

Although our study provides comprehensive pan-cancer evidence supporting the oncogenic role of TRPV1 and its potential as an immunomodulatory biomarker, several limitations should be acknowledged. First, the functional validation was restricted to only two cancer types (head and neck and lung cancers); whether TRPV1 exerts similar effects in other malignancies remains unverified. Second, while bioinformatic analyses revealed strong correlations between TRPV1 expression, immune cell infiltration, and immune checkpoint levels, these associations do not establish causality. Third, the mechanistic insights—particularly regarding TRPV1’s regulation of the PI3K/AKT pathway, autophagy, and immune modulation—are primarily based on in vitro experiments, lacking in vivo confirmation or clinical correlation. Finally, the therapeutic potential of targeting TRPV1 has not been tested in preclinical models or patient-derived samples, limiting immediate translational applicability. Future studies integrating multi-omics data, animal models, and clinical cohorts are needed to fully elucidate TRPV1’s context-dependent roles and its feasibility as a therapeutic target.

## Conclusion

In conclusion, our pan-cancer analysis demonstrates that TRPV1 is frequently upregulated across multiple malignancies and is significantly associated with poor prognosis, immune cell infiltration, and immune checkpoint expression. Functional experiments in head and neck and lung cancer models suggest that TRPV1 promotes cancer cell migration, potentially through modulation of the PI3K/AKT pathway, autophagy, and the tumor immune microenvironment. These findings collectively indicate that TRPV1 may serve not only as a prognostic biomarker but also as a potential immunomodulatory target in cancer therapy. Further mechanistic and translational studies are warranted to explore its therapeutic utility across diverse cancer types.

## Supplementary Information

Supplementary Material 1.

Supplementary Material 2.

Supplementary Material 3.

Supplementary Material 4.

Supplementary Material 5.

## Data Availability

Data are provided within the manuscript or supplementary information files, and any additional data related to this study are available from the corresponding author upon reasonable request.

## Abbreviations

TRPV1: Transient receptor potential vanilloid 1
ICI: Immune check-point
TME: Tumor microenvironment
TCGA: The Cancer Genome Atlas
GTEx: Genotype-Tissue Expression
DEGs: Differential expression genes
OS: Overall survival
DFS: Disease-free survival
TISCH: Tumor Immune Single-cell Hub
MSI: Microsatellite instability
TMB: Tumor mutation burden
PVDF: Polyvinylidene fluoride
ATCC: American Type Culture Collection
DMEM: Dulbecco’s Modified Eagle Medium
SD: Standard error

## References

[1] Bray F, Laversanne M, Weiderpass E, Soerjomataram I. The ever-increasing importance of cancer as a leading cause of premature death worldwide. Cancer. 2021;127:3029–30. 34086348 10.1002/cncr.33587

[2] Sung H, Ferlay J, Siegel RL, Laversanne M, Soerjomataram I, Jemal A, et al. Global Cancer Statistics 2020: GLOBOCAN Estimates of Incidence and Mortality Worldwide for 36 Cancers in 185 Countries, CA: Cancer J Clin. 2021;71:209– 49. 10.3322/caac.21660 33538338

[3] Huis In ‘t Veld RV, Ma S, Kines RC, Savinainen A, Rich C, Ossendorp F, et al. Immune checkpoint inhibition combined with targeted therapy using a novel virus-like drug conjugate induces complete responses in a murine model of local and distant tumors. Cancer Immunol, Immunother: CII. 2023;72:2405-22. 10.1007/s00262-023-03425-3 PMC10264500 36997666

[4] Hargadon KM, Johnson CE, Williams CJ. Immune checkpoint Blockade therapy for cancer: an overview of FDA-approved immune checkpoint inhibitors. Int Immunopharmacol. 2018;62:29–39. 29990692 10.1016/j.intimp.2018.06.001

[5] Janiczek M, Szylberg Ł, Kasperska A, Kowalewski A, Parol M, Antosik P, et al. Immunotherapy as a promising treatment for prostate cancer: A systematic review. J Immunol Res. 2017;2017:4861570. 29109964 10.1155/2017/4861570 PMC5646317

[6] Roccuzzo G, Moirano G, Fava P, Maule M, Ribero S, Quaglino P. Obesity and immune-checkpoint inhibitors in advanced melanoma: A meta-analysis of survival outcomes from clinical studies. Sem Cancer Biol. 2023;91:27–34. 10.1016/j.semcancer.2023.02.010 36871633

[7] Nie Y, Feng F, Luo W, Sanders AJ, Zhang Y, Liang J, et al. Overexpressed transient receptor potential vanilloid 1 (TRPV1) in lung adenocarcinoma harbours a new opportunity for therapeutic targeting. Cancer Gene Ther. 2022;29:1405–17. 35354949 10.1038/s41417-022-00459-0 PMC9576597

[8] Kapoor-Narula U, Lenka N. Cancer stem cells and tumor heterogeneity: Deciphering the role in tumor progression and metastasis. Cytokine. 2022;157:155968. 35872504 10.1016/j.cyto.2022.155968

[9] Guan XY, Guan XL, Jiao ZY. Improving therapeutic resistance: beginning with targeting the tumor microenvironment. J Chemother. 2022;34:492–516. 34873999 10.1080/1120009X.2021.2011661

[10] Zhang W, Li S, Li C, Li T, Huang Y. Remodeling tumor microenvironment with natural products to overcome drug resistance. Front Immunol. 2022;13:1051998. 36439106 10.3389/fimmu.2022.1051998 PMC9685561

[11] Junttila MR, de Sauvage FJ. Influence of tumour micro-environment heterogeneity on therapeutic response. Nature. 2013;501:346–54. 24048067 10.1038/nature12626

[12] Qian BZ, Pollard JW. Macrophage diversity enhances tumor progression and metastasis. Cell. 2010;141:39–51. 20371344 10.1016/j.cell.2010.03.014 PMC4994190

[13] Kalluri R, Zeisberg M. Fibroblasts in cancer. Nat Rev Cancer. 2006;6:392–401. 16572188 10.1038/nrc1877

[14] Whiteside TL. The tumor microenvironment and its role in promoting tumor growth. Oncogene. 2008;27:5904–12. 18836471 10.1038/onc.2008.271 PMC3689267

[15] Zeng D, Li M, Zhou R, Zhang J, Sun H, Shi M, et al. Tumor microenvironment characterization in gastric cancer identifies prognostic and immunotherapeutically relevant gene signatures. Cancer Immunol Res. 2019;7:737–50. 30842092 10.1158/2326-6066.CIR-18-0436

[16] Xia R, Dekermendjian K, Lullau E, Dekker N. TRPV1: a therapy target that attracts the pharmaceutical interests. Adv Exp Med Biol. 2011;704:637–65. 21290320 10.1007/978-94-007-0265-3_34

[17] Aghazadeh Tabrizi M, Baraldi PG, Baraldi S, Gessi S, Merighi S, Borea PA. Medicinal Chemistry, Pharmacology, and clinical implications of TRPV1 receptor antagonists. Med Res Rev. 2017;37:936–83. 27976413 10.1002/med.21427

[18] Li L, Chen C, Chiang C, Xiao T, Chen Y, Zhao Y, et al. The impact of TRPV1 on cancer pathogenesis and therapy: A systematic review. Int J Biol Sci. 2021;17:2034–49. 34131404 10.7150/ijbs.59918 PMC8193258

[19] Zhai K, Liskova A, Kubatka P, Büsselberg D. Calcium entry through TRPV1: a potential target for the regulation of proliferation and apoptosis in cancerous and healthy cells. Int J Mol Sci. 2020;21:4177. 10.3390/ijms21114177 PMC7312732 32545311

[20] Tang Z, Kang B, Li C, Chen T, Zhang Z. GEPIA2: an enhanced web server for large-scale expression profiling and interactive analysis. Nucleic Acids Res. 2019;47:W556–60. 31114875 10.1093/nar/gkz430 PMC6602440

[21] Sun D, Wang J, Han Y, Dong X, Ge J, Zheng R, et al. TISCH: a comprehensive web resource enabling interactive single-cell transcriptome visualization of tumor microenvironment. Nucleic Acids Res. 2021;49:D1420–30. 33179754 10.1093/nar/gkaa1020 PMC7778907

[22] Li T, Fan J, Wang B, Traugh N, Chen Q, Liu JS, et al. TIMER: A web server for comprehensive analysis of Tumor-Infiltrating immune cells. Cancer Res. 2017;77:e108–10. 29092952 10.1158/0008-5472.CAN-17-0307 PMC6042652

[23] Li X, Zeng S, Ding Y, Nie Y, Yang M. Comprehensive analysis of the potential Immune-Related biomarker transporter associated with antigen processing 1 that inhibits metastasis and invasion of ovarian cancer cells. Front Mol Biosci. 2021;8:763958. 34957213 10.3389/fmolb.2021.763958 PMC8702961

[24] Linch SN, Redmond WL. How do I steer this thing? Using dendritic cell targeted vaccination to more effectively guide the antitumor immune response with combination immunotherapy. J Immunother Cancer. 2016;4:31. 27330804 10.1186/s40425-016-0135-z PMC4915175

[25] Wolchok JD, Kluger H, Callahan MK, Postow MA, Rizvi NA, Lesokhin AM, et al. Nivolumab plus ipilimumab in advanced melanoma. N Engl J Med. 2013;369:122–33. 23724867 10.1056/NEJMoa1302369 PMC5698004

[26] Ribas A, Wolchok JD. Cancer immunotherapy using checkpoint blockade, Science. 2018;359:1350–5. 10.1126/science.aar4060 PMC7391259 29567705

[27] Hassani N, Jafari-Gharabaghlou D, Dadashpour M, Zarghami N. The effect of dual bioactive compounds Artemisinin and Metformin Co-loaded in PLGA-PEG Nano-particles on breast cancer cell lines: potential apoptotic and Anti-proliferative action. Appl Biochem Biotechnol. 2022;194:4930–45. 35674922 10.1007/s12010-022-04000-9

[28] Pourgholi A, Dadashpour M, Mousapour A, Firouzi Amandi A, Zarghami N. Anticancer potential of Silibinin loaded polymeric nanoparticles against breast cancer cells: insight into the apoptotic genes targets. Asian Pac J Cancer Prev. 2021;22:2587–96. 34452574 10.31557/APJCP.2021.22.8.2587 PMC8629447

[29] Oh SJ, Lim JY, Son MK, Ahn JH, Song KH, Lee HJ, et al. TRPV1 Inhibition overcomes cisplatin resistance by blocking autophagy-mediated hyperactivation of EGFR signaling pathway. Nat Commun. 2023;14:2691. 37165076 10.1038/s41467-023-38318-7 PMC10172196

[30] Gao N, Yang F, Chen S, Wan H, Zhao X, Dong H. The role of TRPV1 ion channels in the suppression of gastric cancer development. J Experimental Clin Cancer Research: CR. 2020;39:206. 10.1186/s13046-020-01707-7 PMC7531167 33008449

[31] Erin N, Szallasi A. Carcinogenesis and Metastasis: Focus on TRPV1-Positive Neurons and Immune Cells. Biomolecules. 2023;13:983. 10.3390/biom13060983 PMC10296534 37371563

[32] Shen C, Fu C, Suo Y, Li K, Zhang Z, Yang S, et al. Pan-cancer analyses of clinical prognosis, immune infiltration, and immunotherapy efficacy for TRPV family using multi-omics data. Heliyon. 2023;9:e16897. 37346342 10.1016/j.heliyon.2023.e16897 PMC10279839

[33] Mariotton J, Cohen E, Zhu A, Auffray C, Barbosa Bomfim CC, Barry Delongchamps N, et al. TRPV1 activation in human Langerhans cells and T cells inhibits mucosal HIV-1 infection via CGRP-dependent and independent mechanisms. Proc Natl Acad Sci USA. 2023;120:e2302509120. 37216549 10.1073/pnas.2302509120 PMC10235960

[34] He LN, Li H, Du W, Fu S, Luo L, Chen T, et al. Machine learning-based risk model incorporating tumor immune and stromal contexture predicts cancer prognosis and immunotherapy efficacy. iScience. 2023;26:107058. 37416452 10.1016/j.isci.2023.107058 PMC10320202

[35] Xu Y, Fu Y, Zhu B, Wang J, Zhang B. Predictive biomarkers of immune checkpoint Inhibitors-Related toxicities. Front Immunol. 2020;11:2023. 33123120 10.3389/fimmu.2020.02023 PMC7572846

[36] Gallois C, Landi M, Taieb J, Sroussi M, Saberzadeh-Ardestani B, Cazelles A, et al. Transcriptomic signatures of MSI-high metastatic colorectal cancer predict efficacy of immune checkpoint Inhibitors, clinical cancer research. an official journal of the American Association for Cancer Research. 2023;39:3771–8. 10.1158/1078-0432.CCR-22-3964 PMC10502457 37439810

[37] Sarkar OS, Donninger H, Al Rayyan N, Chew LC, Stamp B, Zhang X, et al. Monocytic MDSCs exhibit superior immune suppression via adenosine and depletion of adenosine improves efficacy of immunotherapy. Sci Adv. 2023;9:eadg3736. 37390211 10.1126/sciadv.adg3736 PMC10313166

