# Supplementary material for: Pan-cancer analysis of TRPV1: a novel immune infiltration-related biomarker for tumor prognosis and immunotherapy response prediction
Source: BMC Cancer. 2026 Jan 14;26:216. doi: 10.1186/s12885-026-15576-4 (PMC12892547; doi:10.1186/s12885-026-15576-4)
Supplement: Supplementary file 1 — Supplementary Material 1. [file 12885_2026_15576_MOESM1_ESM.docx]

**
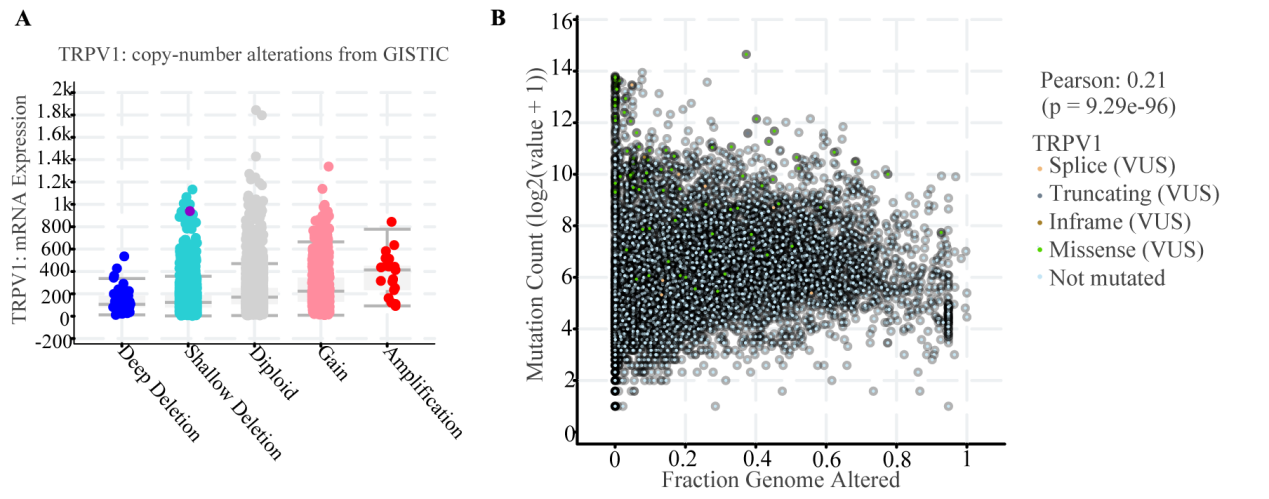
**

**Figure S1. The genomic alteration of TRPV1 in cancers.** (**A**) Copy number alterations from GISTIC. (**B**) Correlation analysis between fraction genome altered and mutation count.


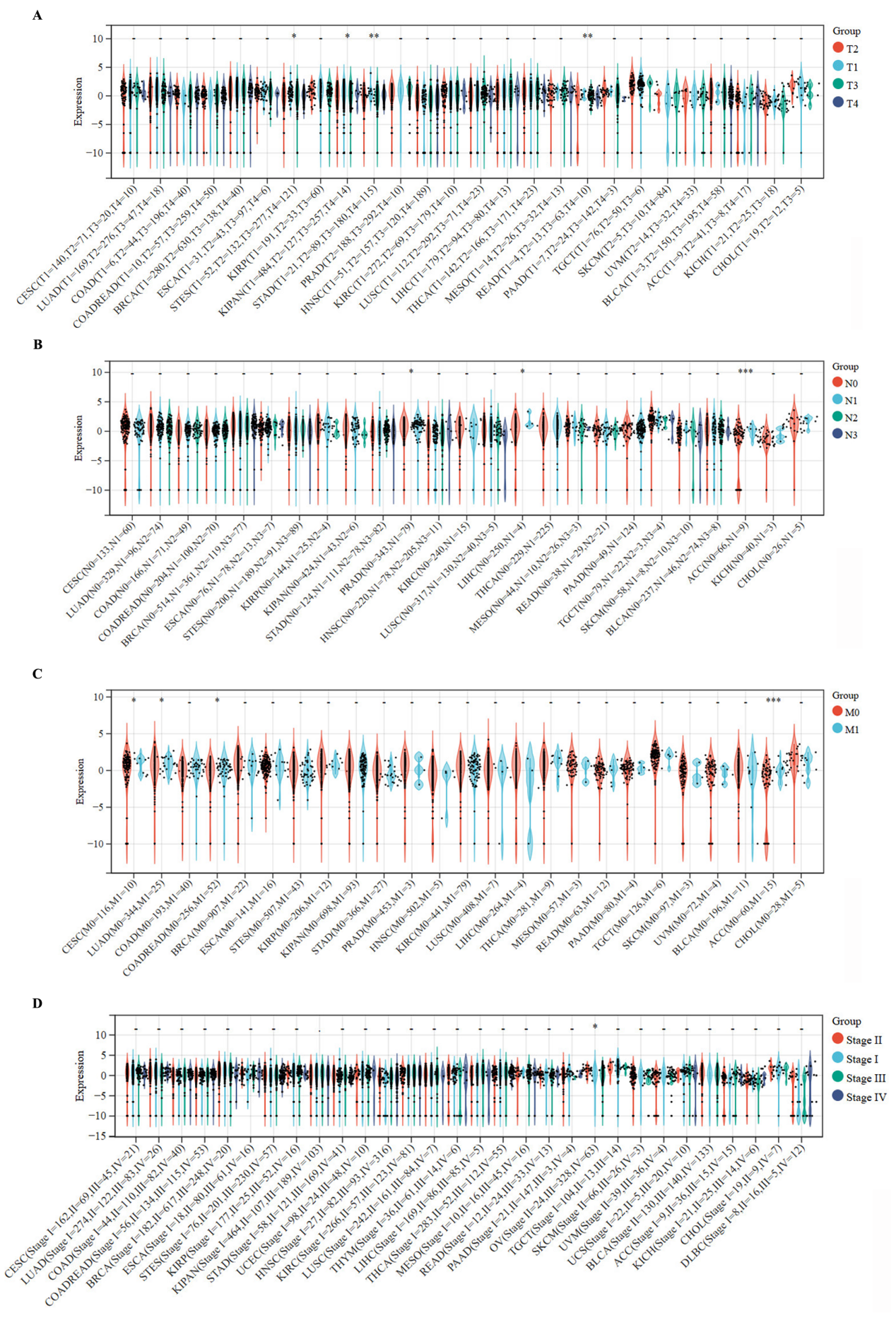


**Figure S2. The expression of TRPV1 in subgroups.** (**A**) TRPV1 in T stage, (**B**) TRPV1 in N stage, (**C**) TRPV1 in M stage, (**D**) TRPV1 in clinical stage.

**
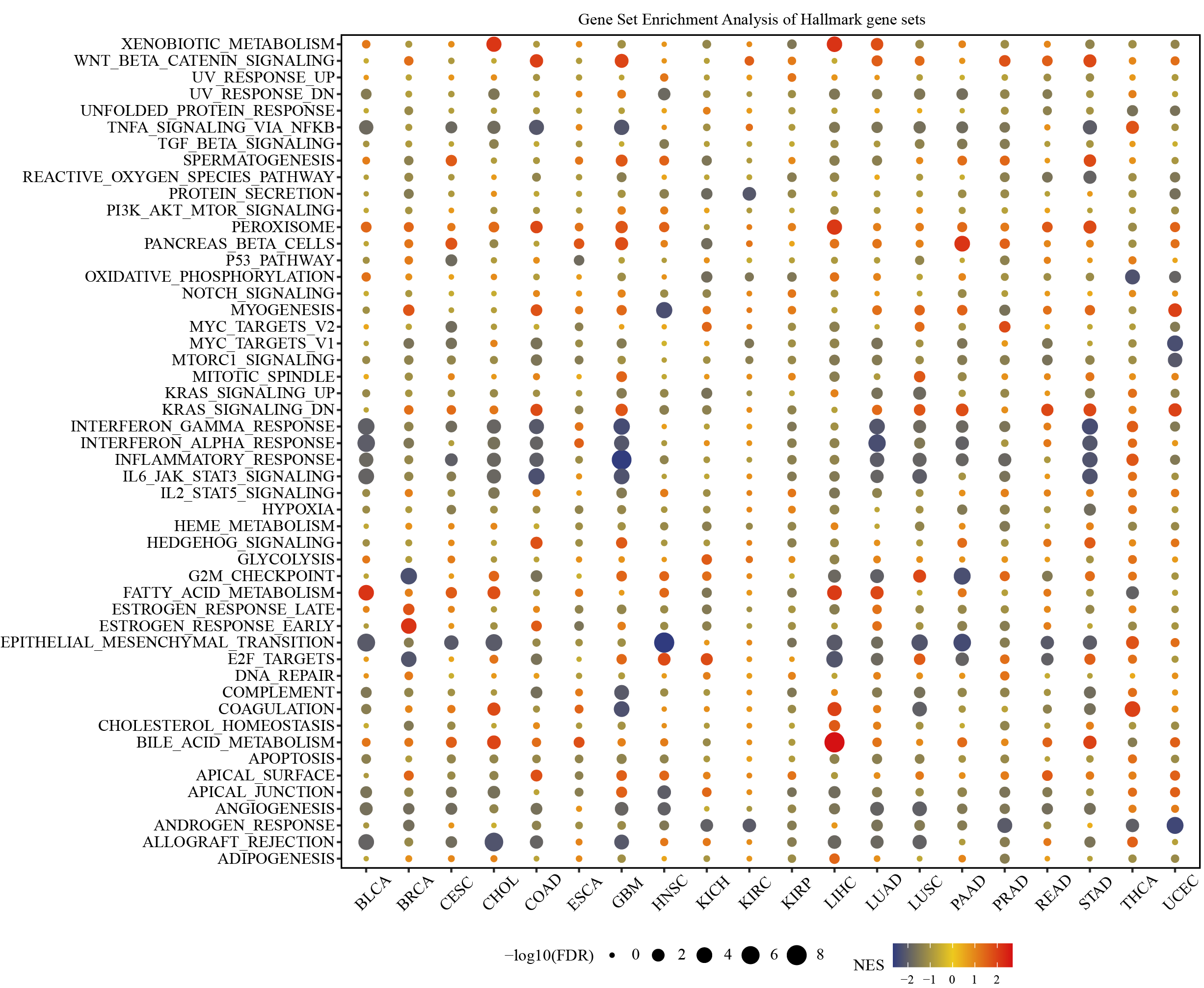
**

**Figure S3. The immune related pathway mediated by** **TRPV1**. Correlations between TRPV1 expression and enriched gene sets in pan-cancer displayed as a bubble plot of NES and log-rank FDR values. NES, normalized enrichment score; FDR, false discovery rate. P < 0.05 and FDR < 0.25 were considered significant.
