# Supplementary figures and images for: Pan-cancer analysis of TRPV1: a novel immune infiltration-related biomarker for tumor prognosis and immunotherapy response prediction
Source: BMC Cancer. 2026 Jan 14;26:216. doi: 10.1186/s12885-026-15576-4 (PMC12892547; doi:10.1186/s12885-026-15576-4)

*
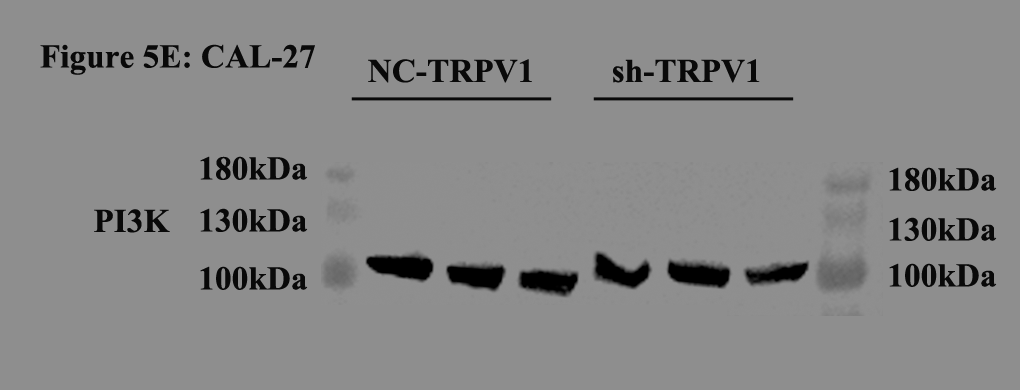
*

*
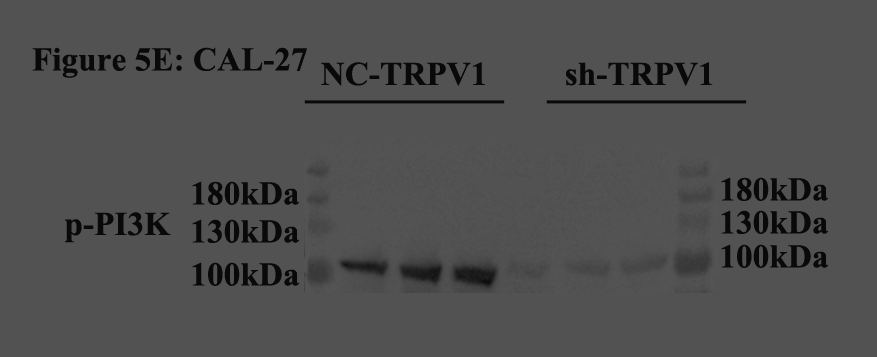
*

*
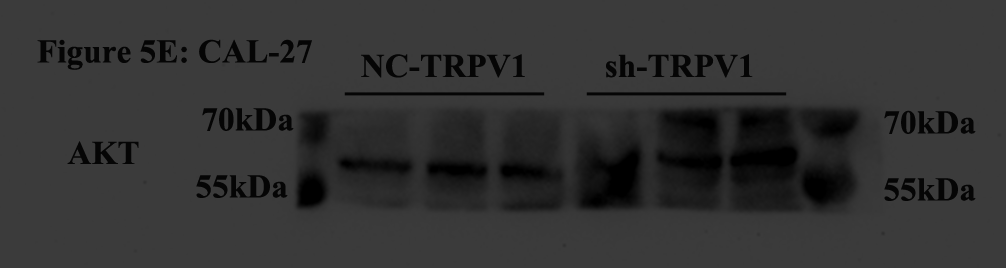
*

*
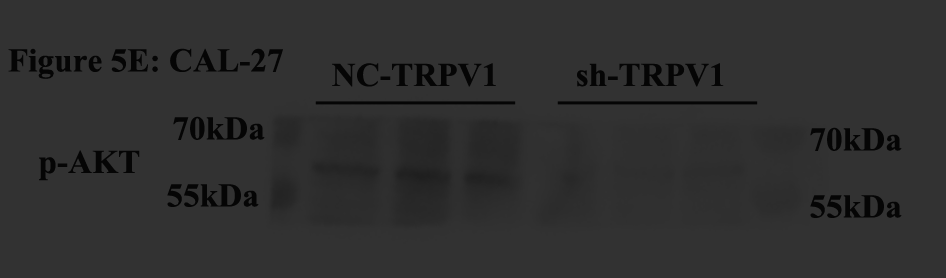
*

*
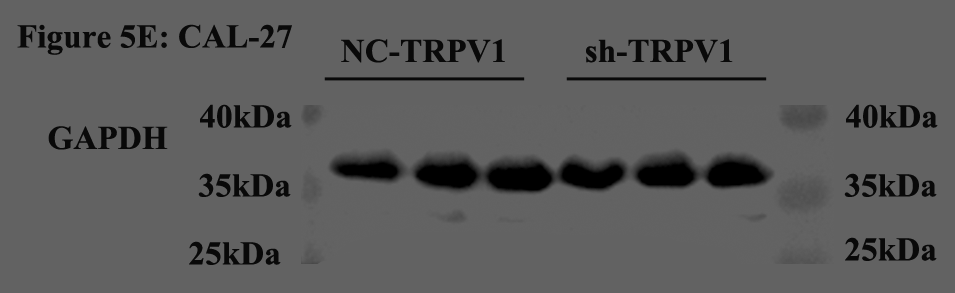
*

*
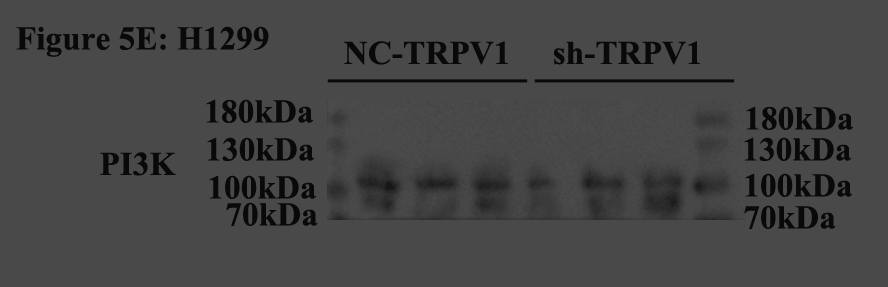
*

*
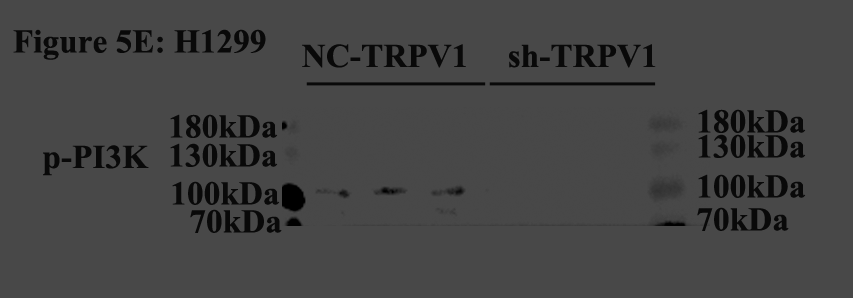
*

*
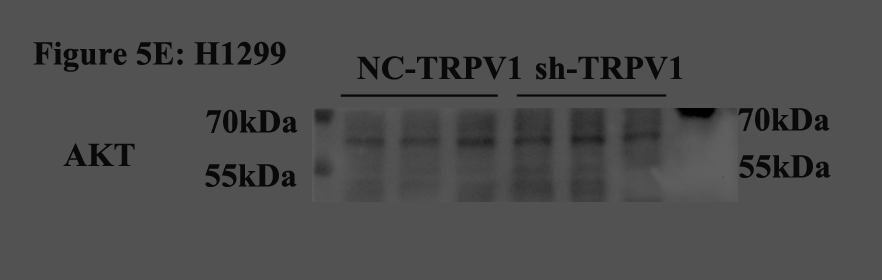
*

*
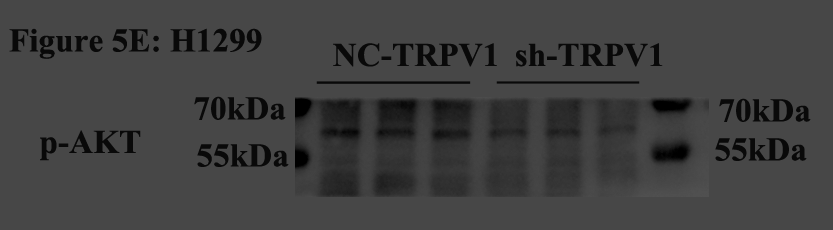
*

*
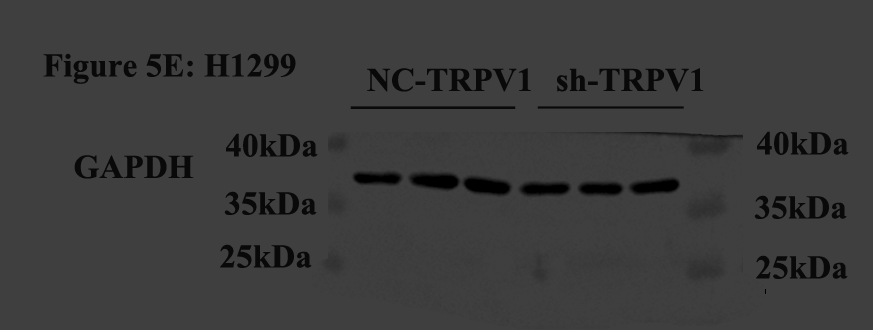
*

*
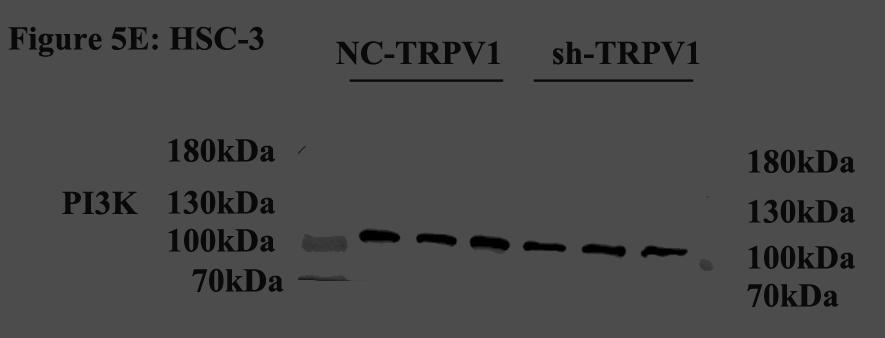
*

*
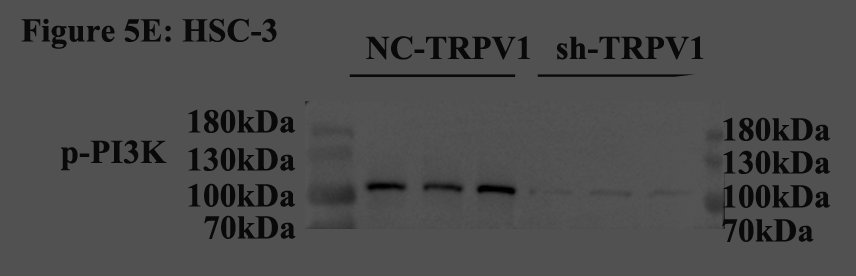
*

*
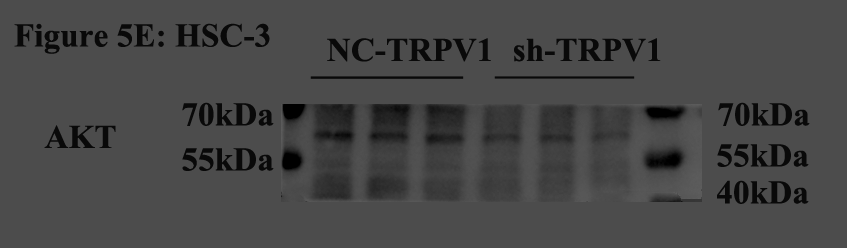
*

*
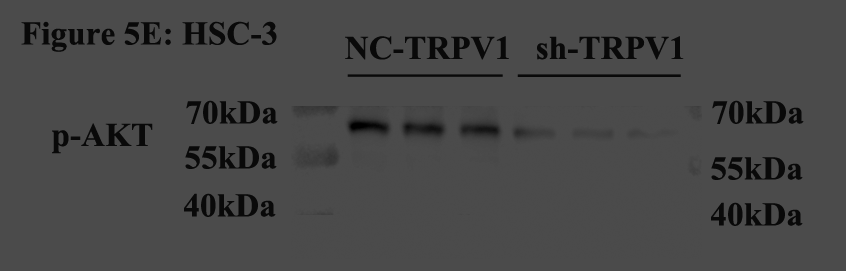
*

*
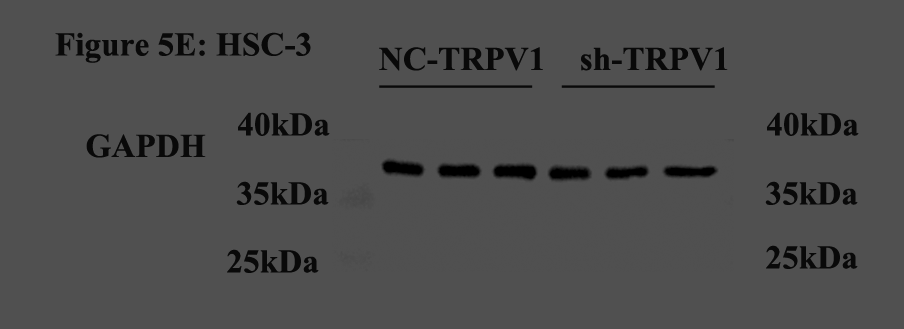
*

*
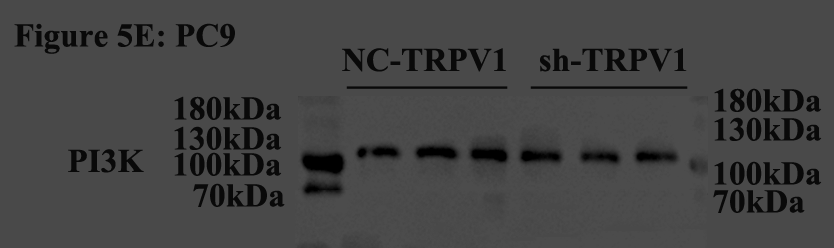
*

*
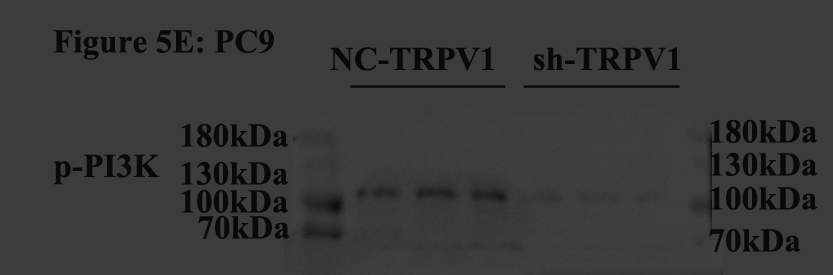
*

*
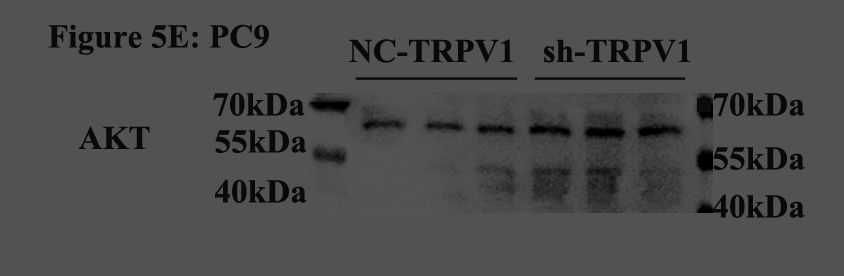
*

*
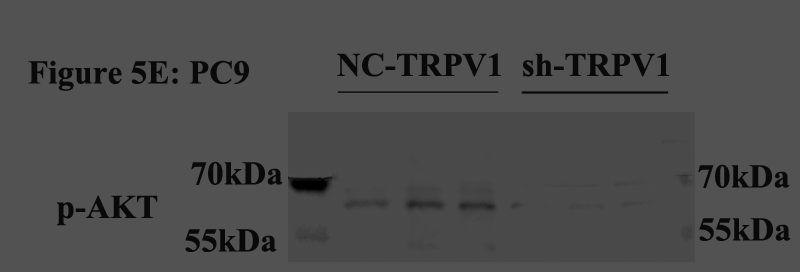
*

*
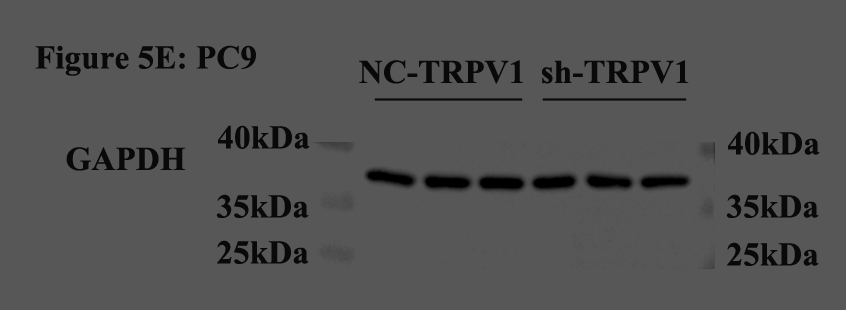
*

Supplement: Supplementary file 5 — Supplementary Material 5. [file 12885_2026_15576_MOESM5_ESM.docx]
